# NSs protein of Schmallenberg virus counteracts the antiviral response of the cell by inhibiting its transcriptional machinery

**DOI:** 10.1099/vir.0.065425-0

**Published:** 2014-08

**Authors:** Gerald Barry, Mariana Varela, Maxime Ratinier, Anne-Lie Blomström, Marco Caporale, Frauke Seehusen, Kerstin Hahn, Esther Schnettler, Wolfgang Baumgärtner, Alain Kohl, Massimo Palmarini

**Affiliations:** 1 MRC–University of Glasgow Centre for Virus Research, Glasgow, UK; 2 Department of Biomedical Sciences and Veterinary Public Health, Swedish University of Agricultural Sciences, P.O. Box 7028, Uppsala SE-750 07, Sweden; 3 Istituto Zooprofilattico Sperimentale dell’Abruzzo e Molise ‘G. Caporale’, Teramo, Italy; 4 Department of Pathology and Center of Systems Neuroscience, University of Veterinary Medicine, Hannover, Germany

## Abstract

Bunyaviruses have evolved a variety of strategies to counteract the antiviral defence systems of mammalian cells. Here we show that the NSs protein of Schmallenberg virus (SBV) induces the degradation of the RPB1 subunit of RNA polymerase II and consequently inhibits global cellular protein synthesis and the antiviral response. In addition, we show that the SBV NSs protein enhances apoptosis *in vitro* and possibly *in vivo*, suggesting that this protein could be involved in SBV pathogenesis in different ways.

Schmallenberg virus (SBV; family *Bunyaviridae*, genus *Orthobunyavirus*) is a virus of ruminants that emerged in 2011 (Hoffmann *et al.*, 2012). SBV causes abortions, stillbirths and malformations in cattle and sheep, and it is believed to spread primarily by biting midges (*Culicoides* spp.) (De Regge *et al.*, 2012; Elbers *et al.*, 2013; Rasmussen *et al.*, 2012; Schnettler *et al.*, 2013). Despite sequence diversity and genus-dependent expression strategies, the NSs proteins of Bunyamwera virus (BUNV) and La Crosse virus (LACV) (both genus *Orthobunyavirus*) and Rift Valley fever virus (genus *Phlebovirus*) inhibit transcription and are considered interferon (IFN) antagonists (Blakqori *et al.*, 2007; Bridgen *et al.*, 2001; Habjan *et al.*, 2009; Verbruggen *et al.*, 2011; Weber *et al.*, 2002). We, and others, have recently shown that the NSs protein of SBV also plays a major role in inhibiting IFN production and that SBV lacking the NSs protein is attenuated *in vivo* (Elliott *et al.*, 2013; Varela *et al.*, 2013). In the current study, we further investigated the roles of the SBV NSs protein.

We previously showed that WT SBV and a NSs deletion mutant (SBVΔNSs) do not differ in multiplication kinetics in a sheep cell line lacking a fully functional IFN system (CPT-Tert) (Arnaud *et al.*, 2010; Varela *et al.*, 2013). Here we performed growth assays of SBV and SBVΔNSs in primary sheep endothelial cells, which have an intact IFN system, and in CPT-Tert and human HEK-293T cells (another weak producer of IFN). The multiplication kinetics of SBVΔNSs in primary endothelial cells was strongly reduced compared with WT SBV, while there was no difference between the viruses in CPT-Tert or HEK-293T cells (Fig. 1a–c).

**Fig. 1.**
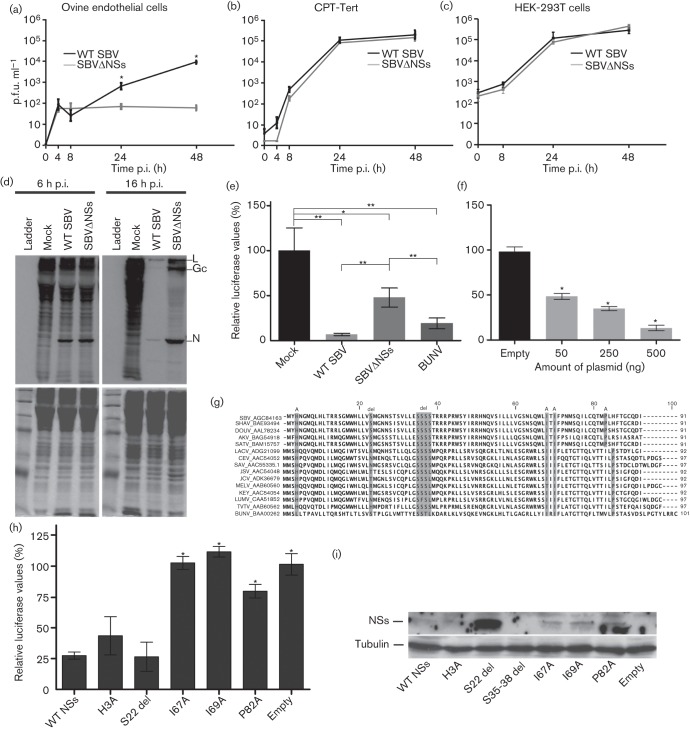
WT SBV (black line) or SBVΔNSs (grey line) growth assays carried out in (a) primary sheep endothelial cells, (b) CPT-Tert cells or (c) HEK-293T cells (m.o.i. 0.01). (d) SDS-PAGE of lysates from ^35^S-methionine-labelled HEK-293T cells infected with WT SBV, SBVΔNSs or mock. Gels were stained with Coomassie blue and then exposed to X-ray films (grey). Positions of SBV proteins polimerase, glycoprotein and nucleocapsid are indicated as ‘L’, ‘Gc’ and ‘N’, respectively. (e) Luciferase activity in HEK-293T cells transfected with a pRL-CMV plasmid (100 ng), infected with WT SBV, SBVΔNSs or BUNV (m.o.i. 1) and then lysed 24 h p.i. Values are relative to mock-infected cells (100 %). (f) Luciferase activity in HEK-293T cells transfected with a pRL-CMV plasmid (100 ng) and either 500 ng of empty plasmid or 50, 250 or 500 ng of a pCI plasmid expressing SBV NSs and then lysed 24 h p.t. (g) Alignment of amino acid residues of the NSs protein of different bunyaviruses. Names on the left of each sequence indicate the name of the virus and the corresponding GenBank accession number. SBV, Schmallenberg virus; SHAV, Shamonda virus; DOUV, Douglas virus; AKV, Akabane virus; SATV, Sathuperi virus; LACV, La Crosse virus; CEV, California encephalitis virus; SAV, San Angelo virus; JSV, Jerry Slough virus; JCV, Jamestown Canyon virus; MELV, Melao virus; KEY, Keystone virus; LUMV, Lumbo virus; TVTV, Trivittatus virus; BUNV, Bunyamwera virus. Highlighted in grey are amino acid residues that were substituted with an alanine (shown as an ‘A’ above the highlighted region) or deleted (‘del’ above the highlighted region). (h) Luciferase activity in HEK-293T cells transfected with pRL-CMV (100 ng) and pCI plasmid (500 ng) expressing either WT SBV NSs, one of the mutant versions of SBV NSs or an empty plasmid and lysed 24 h p.t. Values are relative to mock-infected cells (100 %). (i) Western blot for SBV NSs and γ-tubulin using lysates from HEK-293T cells transfected with pRL-CMV (100 ng) and pCI plasmid (500 ng) expressing WT NSs or one of the mutant versions of NSs shown in (g). Cells were treated with MG132 (10 µM) 8 h p.t. and lysed 24 h p.t. Note that expression of the deletion mutant S35-38 del could not be confirmed and consequently was not used further. All assays shown in this figure were performed a minimum of three times and each assay was performed in triplicate. Statistically significant differences are indicated by asterisks (*P<0.05, **P<0.01; one-way ANOVA). Error bars indicate SD.

To test if SBV could block host-cell gene and protein expression by suppressing transcription, translation or both (which would consequently inhibit a strong antiviral response), we first infected HEK-293T cells with WT SBV or SBVΔNSs at an m.o.i. of 1 and monitored nascent protein synthesis by ^35^S-methionine (PerkinElmer) labelling (0.8 MBq ml^−1^). We labelled cells for 2 h beginning at either 6 h or 16 h post-infection (p.i.) (before the appearance of cytopathic effect). Cells were then lysed, and lysates were subjected to SDS-PAGE. Protein synthesis was decreased in both WT SBV- and SBVΔNSs-infected cells, although this effect appeared less pronounced in the latter (Fig. 1d) suggesting that NSs contributes to the inhibition of host-cell gene expression in SBV-infected cells. Similar results were seen in CPT-Tert cells (not shown). Interestingly, the virus protein bands at 16 h (bands ‘L’, ‘Gc’ and ‘N’ on the gel shown in Fig. 1d) were more prominent in SBVΔNSs-infected cells. The reasons for this are unclear, but it could suggest that NSs plays a regulatory/inhibitory role in the expression of other SBV proteins. This phenomenon has also been observed in BUNV infections (Bridgen *et al.*, 2001).

To confirm these results, we used a luciferase-based assay to quantify the inhibition of gene expression. HEK-293T cells were transfected with pRL-CMV (Promega), a *Renilla* luciferase-expressing plasmid. Cells were then infected with WT SBV, SBVΔNSs, BUNV (positive control) (Léonard *et al.*, 2006) (m.o.i. 1) or mock at 16 h post-transfection (p.t.), and luciferase activity was measured 24 h p.i. using the *Renilla*-Glo Luciferase Assay System (Promega). Relative luciferase values in cells infected by SBV and BUNV were 91 % and 75 %, respectively, lower than in mock-infected cells. In contrast, relative luciferase values in SBVΔNSs-infected cells were only 45 % lower than in mock-infected cells (Fig. 1e). Similar results were seen in CPT-Tert cells (not shown). These data confirmed that SBV NSs significantly inhibits host-cell gene expression. Importantly, we found a dose-dependent block of luciferase expression in cells transfected simultaneously with varying amounts of a pCI plasmid expressing the SBV NSs protein and a fixed amount of the pRL-CMV plasmid (100 ng), indicating that SBV NSs alone can inhibit cellular protein production (Fig. 1f).

The structure of the NSs protein of SBV (or of any other bunyavirus) has not been solved. To understand what domains of the protein are involved in inhibiting host-cell gene expression, we introduced several deletions or substitutions in regions that are highly conserved across a wide range of bunyaviruses (Fig. 1g). WT or mutant NSs expression plasmids (500 ng) were transfected into HEK-293T cells along with the pRL-CMV plasmid (100 ng), and luciferase activity was measured 24 h p.t. (Fig. 1h). Substitutions or deletions in the N-terminal region of SBV NSs (H3A, S22del) had no effect on the NSs protein’s ability to inhibit luciferase expression. In contrast, substitutions in the C-terminal region (167A, 169A and P82A) significantly impaired NSs protein activity. We used Western blotting to confirm expression of the mutant NSs proteins in transfected HEK-293T cells (Fig. 1i) using an antiserum against SBV NSs (GenScript). Expression, even of WT SBV NSs, was difficult to detect with our reagents. The NSs proteins of other bunyaviruses are known to be rapidly degraded (Simons *et al.*, 1992; van Knippenberg *et al.*, 2013; Vialat *et al.*, 2000). Thus, the addition of the proteosome inhibitor MG132 (10 µM) to the transfected cells and high exposure times of the membranes during blotting were required to visualize NSs expression (although the signal was weak). These results suggest that the C-terminal region of SBV NSs is critical for its inhibitory function, similar to that in the BUNV NSs protein (Léonard *et al.*, 2006). Interestingly, the NSs S22 del mutant showed a high level of expression. The reason for this is unclear but this mutation may stabilize NSs, thus reducing its degradation in a similar fashion to a mutation recently shown in BUNV NSs (van Knippenberg *et al.*, 2013).

Data from other bunyaviruses suggest that NSs may reduce gene expression by targeting cellular RNA synthesis (Habjan *et al.*, 2009; Léonard *et al.*, 2006; Weber *et al.*, 2002). A Click-iT RNA assay (Life Technologies) was used to detect newly synthesized RNA in HEK-293T cells infected with WT SBV or SBVΔNSs. This assay involves labelling newly made RNA with a modified nucleoside, 5-ethynyl uridine. Cells are then fixed and a fluorescently labelled molecule is ligated to the modified nucleoside. This fluorescence can then be visualized by microscopy (Jao & Salic, 2008). RNA levels in WT SBV-infected cells were markedly lower than in SBVΔNSs-infected cells or uninfected cells, suggesting that the SBV NSs protein reduces cellular RNA levels (Fig. 2a).

**Fig. 2.**
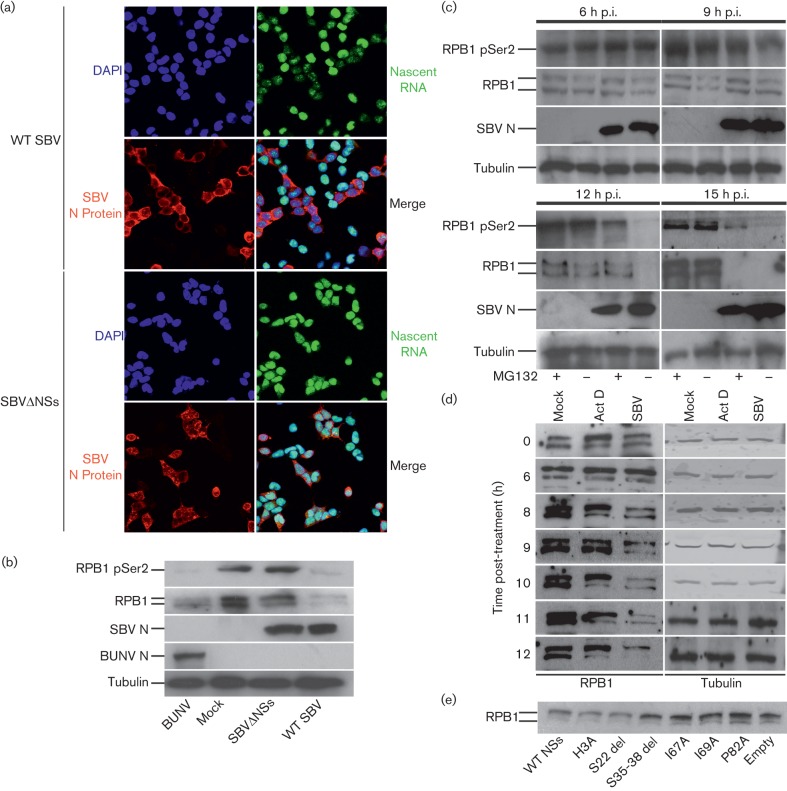
(a) Newly synthesized RNA was labelled in WT SBV- or SBVΔNSs-infected HEK-293T (16 h p.i.); cells were also immunolabelled for the SBV N protein (DAPI, blue; RNA, green; SBV N protein, red). (b) HEK-293T cells were infected with WT SBV, SBVΔNSs, BUNV (m.o.i. 1) or mock. Cell lysates (24 h p.i.) were analysed by Western blot for SBV N protein, BUNV N protein, RPB1, RPB1 pSer2 and γ-tubulin. (c) Western blot for RPB1, RPB1 pSer2, SBV N protein and γ-tubulin, using lysates from cells treated or not treated with MG132 and infected with WT SBV (m.o.i. 1) or mock at the indicated time points. (d) Western blot for RPB1 and γ-tubulin using lysates from HEK-293T cells infected with WT SBV (m.o.i. 1), treated with actinomycin D (Act D, 1 µg ml^−1^) or mock-treated. (e) Western blot for RPB1, using lysates from HEK-293T cells transfected with pRL-CMV (100 ng) and pCI plasmid (500 ng) expressing WT NSs or one of the mutant versions of NSs outlined in Fig. 1 treated with MG132 (10 µM) 8 h p.t. and lysed 24 h p.t. These are the same samples as shown in Fig. 1(i); hence, the γ-tubulin loading control is shown in Fig. 1(i).

BUNV NSs blocks the phosphorylation of the C terminus of the RPB1 subunit of RNA polymerase II (RNAP II) (Léonard *et al.*, 2006; Thomas *et al.*, 2004; van Knippenberg *et al.*, 2010). In contrast, LACV NSs triggers the degradation of RPB1 (Verbruggen *et al.*, 2011). To examine whether SBV NSs targets the RNAP II complex, we infected HEK-293T cells with WT SBV, SBVΔNSs or BUNV (m.o.i. 1) and analysed cell lysates 24 h p.i. by Western blot using antibodies specific for RPB1 (Santa Cruz), a phosphorylated form of RPB1 (RPB1 pSer2, Covance), SBV N protein (Varela *et al.*, 2013), BUNV N protein (Proteintech Group) and γ-tubulin (Sigma). While RPB1 expression was undetectable in BUNV- and SBV-infected cells, expression remained strong in SBVΔNSs-infected cells (Fig. 2b). However, it was unclear whether SBV NSs caused degradation of the RPB1 protein similarly to LACV NSs or inhibited phosphorylation (and thus indirectly allowed RPB1 degradation) like BUNV. Therefore, HEK-293T cells were infected with WT SBV (m.o.i. 1) and grown with or without the proteasome inhibitor MG132 (10 µM). We found that RPB1 was degraded over time (6–15 h p.i.), but this degradation was delayed by MG132. RPB1 pSer2 disappeared along with RPB1, suggesting that SBV NSs targets RPB1 for proteasomal degradation rather than dephosphorylation (Fig. 2c).

RPB1 has a half-life of approximately 6 h in mammalian cells (Akhrymuk *et al.*, 2012). The loss of RPB1 could be a direct result of NSs-induced degradation or an indirect global effect on cellular transcription. To evaluate these two possibilities, we assessed RPB1 expression in cells treated with actinomycin D (a transcription inhibitor) or infected with WT SBV (m.o.i. 1) at different times post-treatment. RPB1 expression decreased dramatically at approximately 9–10 h p.i., whereas it persisted until at least 12 h post-actinomycin D treatment (Fig. 2d). These data strongly suggest that the loss of RPB1 is directly induced by SBV NSs expression and not the indirect result of inhibition of cellular transcription. Crucially, by measuring expression in the luciferase assay and via comparison with RPB1 expression in control cells transfected with empty plasmids, we detected a reduction in RPB1 levels in cells transfected with expression plasmids for SBV NSs or with the NSs mutants H3A and S22 del. On the other hand, the levels of RBP1 in cells transfected with expression plasmids for those NSs mutants that did not show any inhibitory activity in luciferase assays (I67A, I69A and P82A) were comparable to those in the control cells (Fig. 2e).

The NSs proteins of many bunyaviruses also affect cellular apoptosis. The NSs protein of BUNV can reduce or delay cell death by counteracting the activity of IRF-3 (Kohl *et al.*, 2003), while LACV NSs appears to enhance apoptosis (Colón-Ramos *et al.*, 2003; Pekosz *et al.*, 1996). To investigate the role of the SBV NSs protein in apoptosis, we infected CPT-Tert cells with WT SBV or SBVΔNSs and measured activated caspase-3/7 in cell lysates using the Caspase-Glo 3/7 Assay kit (Promega). The amount of activated caspase-3/7 in WT SBV–infected cells was significantly higher than in mock- or SBVΔNSs-infected cells (Fig. 3a). Importantly, caspase-3/7 activation slightly but significantly increased in cells transfected with a plasmid expressing the SBV NSs protein compared with activation in cells transfected with empty plasmid (Fig. 3b). Similar results were observed in HEK-293T cells (not shown). Thus, it appears that similar to LACV NSs, SBV NSs enhances the rate of apoptotic cell death. To assess the relevance of these observations *in vivo*, we infected newborn NIH-Swiss mice intracerebrally with WT SBV or SBVΔNSs virus (400 p.f.u., two mice per group per time point). Brains were collected at 48, 72, 96 and 120 h post-inoculation and stained for the presence of SBV N protein and activated caspase-3 (Cell Signalling) by immunohistochemistry. Cells positive for either activated caspase-3 or SBV N protein were counted in different fields, in three different areas of the brain (cerebellum, cerebrum and brainstem), and the mean number of positive cells per mm^2^ was recorded. Although differences were not statistically significant, brain sections collected from mice infected with WT SBV contained a higher proportion of caspase-3-positive cells than those infected with SBVΔNSs, supporting a role for the SBV NSs protein in apoptosis (Fig. 3c, d).

**Fig. 3.**
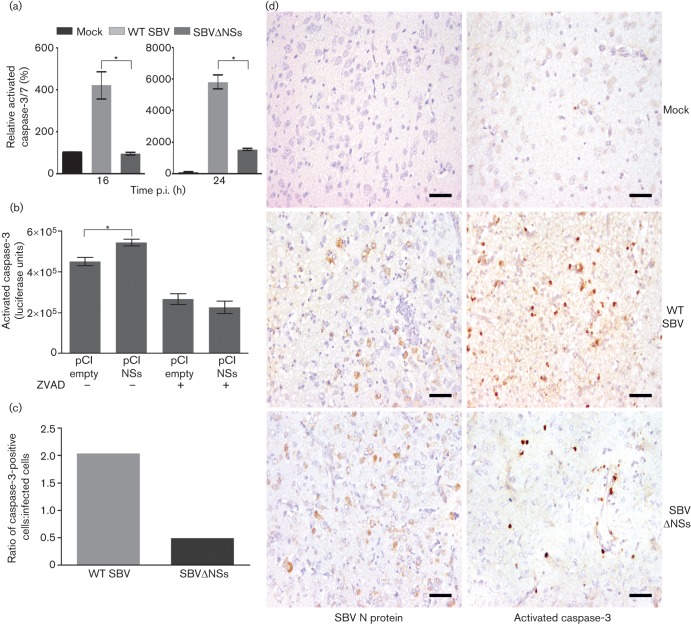
(a) Level of activated caspase-3/7 in CPT-Tert cells infected (m.o.i. 1) with WT SBV, SBVΔNSs or mock (24 h p.i.). (b) Level of activated caspase-3/7 in CPT-Tert cells transfected with a pCI plasmid (100 ng) expressing SBV NSs or an empty pCI plasmid and then treated with Z-VAD-FMK (ZVAD, 20 µM) or mock (24 h p.t.). Both assays (in a and b) were carried out three times in triplicate. Statistically significant differences are indicated by asterisks (*P*<0.05, one-way ANOVA). Bars indicate SD. (c) Ratio of the total number of caspase-3-positive cells to infected cells per mm^2^ of brains from NIH-Swiss mice that were inoculated intracerebrally with WT SBV, SBVΔNSs or medium. (d) Representative micrographs showing caspase-3-positive and SBV-positive cells (brown staining) as revealed by immunohistochemistry of mouse brain sections (brainstem).

In conclusion, our data provide insight into the functions of the NSs protein of a newly emerged bunyavirus of ruminants. The SBV NSs protein targets the RPB1 subunit of RNAP II for degradation, preventing transcription in infected cells and consequently *de novo* protein synthesis. Inhibition of cellular transcription leads to a reduction in IFN production that favours SBV replication and spread and thus contributes to pathogenesis. Indeed, replication of SBVΔNSs is grossly impaired in IFN-competent primary sheep cells, while it is identical to WT SBV in IFN-incompetent cell lines, showing that virus targeting of the cellular transcriptional machinery is essential to overcome an intact antiviral response. Finally, we found evidence supporting a pro-apoptotic role for SBV NSs protein (similar to LACV NSs, but in contrast to BUNV NSs), which may also contribute to viral pathogenesis.
